# Self-management practice, associated factors and its relationship with health literacy and patient activation among multi-morbid COPD patients from rural Nepal

**DOI:** 10.1186/s12889-020-8404-7

**Published:** 2020-03-06

**Authors:** Uday Narayan Yadav, Jane Lloyd, Hassan Hosseinzadeh, Kedar Prasad Baral, Narendra Bhatta, Mark Fort Harris

**Affiliations:** 1 Centre for Primary Health Care and Equity, UNSW, Sydney, Australia; 2 Forum for Health Research and Development, Dharan, Nepal; 3 The University of Wollongong, Sydney, Australia; 4 School of Public Health, Patan Academy of Health Sciences, Kathmandu, Nepal; 5 Department of Pulmonary, Critical Care and Sleep Medicine, B.P. Koirala Institute of Health Sciences (BPKIHS), Dharan, Nepal

**Keywords:** Health literacy, Multi-morbid Chronic Obstructive Pulmonary Disease (COPD), Patient activation, Self-management practices

## Abstract

**Background:**

Chronic Obstructive Pulmonary Disease (COPD) is a progressive and debilitating condition that affects individuals’ quality of life. COPD self-management and supports provided by carers is key to the quality of life people living with COPD. Health literacy (HL) and Patient Activation (PA) are main drivers of self-management practices (SMPs). However, their contribution remains to be fully explored. This study aimed to examine the level of self-management practices, and the relationship with socio-demographic factors, HL and PA among multi-morbid COPD patients from rural Nepal.

**Methods:**

This is a cross-sectional study conducted between July 2018 and January 2019. Patients completed a survey, including Self-management Practices questionnaire (SMPQ), five domains of the Health Literacy Questionnaire (HLQ), and Patient Activation Measure (PAM). The relationship between HL, PAM, and SMPs was examined using univariate statistics. Multivariable analysis was conducted to identify the factors associated with SMPs.

**Results:**

A total of 238 patients responded to the study. The mean score of SMPQ was 45.31(SD = 9.00). The HLQ and PAM scores were positively correlated with the total score of SMPQ. Low level of SMPs were found to be positively associated with being uneducated (β = − 0.43, *p* = .001), having a low family income (β = − 5.22, *p* = .002), and, negatively associated with the presence of more than one co-morbidity (β = 3.58, *p* = 0.007) after controlling for other socio-demographic variables in the multivariable analysis.

**Conclusion:**

The overall SMPs among this sample of Nepalese with COPD were low. Our findings highlight the need to implement a self-management intervention program involving patient activation and health literacy-focused activities for COPD, creating a support system for patients from low-income families and low education.

## Background

Chronic Obstructive Pulmonary Disease (COPD) is a progressive and debilitating condition that impairs the quality of life of patients preventing participation in daily living activities because of progressively worsening symptoms. The presence of two or more other chronic conditions among COPD patients increases the severity of the condition, posing significant health challenges to patients’ health and health systems [1]. The World Health Organisation (WHO) data shows the condition of COPD patients to be much worse in developing countries, where more than 90% of deaths occur because of lack of access to care and actionable strategies for prevention and management of COPD, than in developed countries [2]. A recent study from Nepal found a COPD prevalence of 11.7% highest among the list of selected non-communicable disease [3]. Despite the great understanding of the pathophysiology of disease and various advances in medicine, COPD remained as an incurable disease. This signifies the need of self-management practices (SMPs) that could play an important role in halting disease progression.

COPD self-management primarily involves self-recognition and management of symptoms, taking medication, having a healthy diet, coping with breathlessness, quitting smoking and engaging in regular physical exercise to maintain good health [4]. Evidence shows that various factors such as educational status [5–7], family history of COPD [6], lack of social support [8] and low socioeconomic status of family [9] are associated with poor SMPs.

Studies that supported patients in self-management have been found to be effective in improving quality of life in COPD patients and reducing hospital admissions [4, 10]. Understanding and using health information to make health decisions [11] and patients being activated to manage their conditions [12], had a positive effect on self-management [13], and improved health outcomes [14–17]. A few studies have demonstrated the association between HL and PA with positive health outcomes [18, 19]. Therefore, role of HL and PA is important in improving quality of life among COPD patients [13]. However, findings are not consistent, with two studies showing a correlation between both patient activation and health literacy [20] with SMPs [21]. However one study found no association between health literacy [22] and SMPs.

The “Right to Health” is stated in the Nepalese constitution [23] and the country has been able to achieve noticeable improvements during the era of Millennium Development Goals (MDGs). Government of Nepal, being a signatory of Sustainable Development Goals (SDGs) is committed in achieving SDGs 3 (sub-goal 3.4) aimed to “reduce one third premature mortality from non-communicable diseases through prevention and treatment with promotion of mental health and well-being” [24]. In this context, although some attention has been paid to SMPs of some chronic diseases such as Diabetes and Hypertension, there has been limited attention to the COPD self-management in Nepal. Therefore, the present study aimed to examine the level of SMPs, and the relationship with socio-demographic factors, HL and PA among multi-morbid COPD patients from rural Nepal.

## Methods

### Setting and study design

Participants were recruited via simple random sampling from the two rural municipalities of *Sunsari district*, Nepal. The study was carried out between July 2018 and January 2019. A random sampling approach was used to select the study participants from the list of 576 study samples obtained from two rural municipalities.

### Sample size

The required sample size of 250 was determined based on following assumptions: prevalence (knowledge on self-management) = 18%, sampling error = 5.0%, CI = 95.0%, and non-response rate = 10.0%. In line with the study protocol, 250 patients were selected randomly from the list of eligible subjects, but, only 238 patients met the eligibility criteria to participate.

To be eligible to participate, study participants had to fulfill the following criteria (i) aged between 18 and 70 years, (ii) diagnosed with COPD with at least one of the co-morbidities such as cardiovascular disease, diabetes, asthma, arthritis, depression, musculoskeletal disorders or gastritis (reflux) on their medical records; and (iii) able to provide consent form. Patients with a hearing disability, severe cognitive disorder, kidney disease, a history of stroke or diagnosed heart attack, and with a terminal illness such as cancer were excluded from the study.

### Measures

Age, gender, ethnicity, education level, marital status, occupation, monthly family income, number of co-morbidities, and self-reported history of alcohol and tobacco use were collected by administering a semi-structured questionnaire.

#### Self-management practices questionnaire (SMPQ)

The 18-item SMPQ was adapted from the 51-item COPD Self-Management Scale (CSMS) [25] based on applicability and acceptability in the Nepalese context. Selected items measured information management, symptoms management, daily lifestyle management, emotional management, and self-efficacy. The responses to each item were graded on 5-point scales where 1 = never, 2 = rarely, 3 = sometimes, 4 = often, and 5 = always.

#### Health literacy

Five domains of the nine domain Health Literacy Questionnaire (HLQ) [26] were selected mainly due to their relevance to the local realities or context of Nepal. The domains included were: (i) feeling understood and supported by healthcare providers (HPS), (ii) having sufficient information to manage my own health (HIS), (iii) social support for health (SS), (iv) ability to find the good health information (AE), and (v) understand the health information well enough to know what to do (UHI) [27]. The responses to each item were graded on 5-point scales.

#### Patient activation

The shortened Nepali version of the PAM (13-item) was used to assess the self-reported knowledge, skill and confidence required for self-management of conditions [28]. PAM score ranges from 0 (no activation) to 100 (high activation) and are divided into four levels as follows: level 1, 0–47; level 2, 48–55; level 3, 56–66; level 4, 67–100 [29].

The English version of the questionnaires were first translated to Nepali and then translated back to English by two public health professionals who were fluent in both Nepali and English language to check the consistency. The Cronbach’s α for SMPQ, HLQ, and PA was above 0.7, indicating good internal consistency.

### Data collection procedure

Interviewer-administered data collection was conducted over 3 months, where data was filled in Nepali questionnaires. The data collection was performed by trained research assistants who were fluent in both Maithili (a local language spoken by chunks in a rural setting) and a Nepali language. Two-day’ hands-on training was provided to the research assistants by the principal investigator of this study group. The principal investigator monitored the entire field activities to ensure data quality and updates were shared with the research team on weekly basis.

### Statistical analyses

Data were analysed using SPSS version 25. Sample characteristics were summarised as frequency and percentage for discrete variables and as means and standard deviation for a continuous variable. Multivariate linear regression analysis was used carried out to identify the factors associated with a low level of self-management practices. Similarly, Spearman’s correlations were carried out to test the relationships of five domains of the HLQ and PAM with self-management practices.

## Results

### Subject characteristics

The characteristics of the study participants are shown in Table 1. Among 238 participants, 55% were women, with a mean age of 59.18 years (SD = 12.19). Most of the participants were uneducated (69.7%) and married (73.9%). Similarly, most participants belonged to higher (Brahmin/Chhetri) caste (41.2%), followed by indigenous (38.2%) and Dalit (20.6%). Most reported Hinduism to be their religion (93.3%). More than half of the study participants were employed as factory workers/laborers. Over 70.0% of the study participants reported a monthly family income of less than NRs. 20,000, (176 USD). More than 80% of the participants were current or past tobacco users. Most of the participants reported the presence of two or more co-morbidities (74.8%).

**Table 1:** Sample characteristics

Characteristics	n	%
Gender		
Male	107	45
Female	131	55
Age category (years)		
≤40	18	7.6
41–55	44	18.5
≥ 56	176	73.9
Education status		
uneducated	166	69.7
educated	72	30.3
Marital status		
Married	176	73.9
Unmarried/divorced/widow/widower	62	26.1
Ethnicity		
Brahmin/Chhetri (Higher caste)	98	41.2
Indigenous (Adivasi Janajati)	91	38.2
Dalits (untouchable caste as per traditional Hindu caste system)	49	20.6
Religion		
Hindu	222	93.3
Islam	16	6.7
Occupation		
Farmer/Housewife	108	45.4
Professional jobs (Business/government jobs/private company)	8	3.4
Factory workers/labourers	122	51.3
Family income level (NRs)		
≲20,000	206	86.6
> 20,000	32	13.4
Use of tobacco product		
No	45	18.9
Yes	193	81.1
Comorbidities		
At least one^a^	60	25.2
Two or more^b^	178	74.8

^a^ Asthma

^b^ combination of any two or more of these diseases: diabetes, cardiovascular disease, depression, arthritis, insomnia, musculoskeletal disorders etc

### Relationship between self-management practices, HL and PA

The mean SMPs score was 45.31(SD = 9.00).

All five of the domains of HL and the PAM score were found to be moderately correlated with a total score of SMPs (Table 2).

**Table 2:** Correlation of HL domains, PAM with total score of SMPs among multi-morbid COPD patients

Variables	Mean ± SD	Spearman’s correlation	*p*-value
PAM	34.18 ± 14.20	.367	< 0.001
HLQ domains			
HPS	1.96 ±.76	.528	< 0.001
HSI	1.56 ±.74	.433	< 0.001
SS	2.73 ±.77	.393	< 0.001
AE	2.02 ±1.10	.406	< 0.001
UHI	1.78 ±.99	.354	< 0.001

*HPS* Feeling understood and supported by healthcare providers, *HIS* Having sufficient information to manage my own health, *SS* Social support for health, *AE* Ability to find the good health information,* UHI* understand the health information well enough to know what to do

### Factors associated with poor SMPs

As shown in Table 3, low levels of SMPs were associated with being uneducated (β = − 0.43, *p* = .001), having a low of family income (β = − 5.22, *p* = .002), and negatively associated with the presence of more than one co-morbidity (β = 3.58, *p* = 0.007) of after controlling for socio-demographic variables in the multivariable analysis.

**Table 3:** Associates of poor SMPs among multi-morbid COPD patients of Nepal

Variables	Unstandardized β	Standardised β	Confidence Interval (CI)	***P***-value
Gender				
Male	Reference	Reference		
Female	0.05	0.003	−2.45 to 2.55	.96
Education status				
Educated	Reference	Reference		
Uneducated	**−0.43**	**−0.22**	**−.7.11to − 1.75**	**.001**
Age group(years)				
≤40	Reference	Reference		
41–55	1.23	0.05	−3.74 to 6.20	.62
≥ 56	2.36	0.11	−2.59 to 7.32	.34
Marital status				
Married	Reference	Reference		
Unmarried/Divorce/Widow/widower	−0.33	−.01	−3.03 to 2.37	.80
Ethnicity				
Higher caste	Reference	Reference		
Indigenous	−0.06	−0.004	−2.54 to 2.41	.95
Dalits (Untouchable as per traditional Hindu caste system)	−.44	−.02	−3.49 to 2.60	.77
Occupation				
Farmer/housewife	Reference	Reference		
Professional jobs (Govt, Private, Businessman)	.97	.02	−5.40 to 7.36	.76
Factory workers/labourers	−1.66	−0.9	−4.08 to .76	.17
Family income level (NRs)#				
< 20,000	**−5.22**	**−.19**	**−8.48 to − 1.96**	**.002**
> 20,001	Reference	Reference		
Use of tobacco products				
No	Reference	Reference		
Yes	−0.97	−.04	−4.30 to 2.35	.56
Presence of chronic illness by comorbidities				
At least one^a^	Reference	Reference		
More than two^b^	**3.58**	**.17**	**.99 to 6.16**	**.007**

^a^ Asthma

^b^ combination of any two or more of these diseases: diabetes, cardiovascular disease, depression, arthritis, insomnia, musculoskeletal disorders etc

Results in bold indicate significant *p*-value at 95% CI

# 1 USD = 110 NRS (Jan 2019 exchange rate)

## Discussion

The objective of this study was to address important gaps in the literature by investigating the level of SMPs, and their relationship with socio-demographic factors, HL and PA among multi-morbid COPD patients from rural Nepal. To our knowledge, this is the first study assessing the level the SMPs among multi-morbid COPD patients living in the rural areas of Nepal.

Our findings showed that participants’ SMPs were very limited. This level was very low compared to studies from China [30], USA [31] and Norway [32]. It is possible that this difference could be due to the difference in the instruments used to measure self-management practices. In this light, a hospital-based study from Nepal reported that 90.7% of COPD patients had a poor level of knowledge on the self-care of COPD [6]. In Nepal, the majority of the COPD patients seek services from the government or private hospitals because of the inadequacy of available services at the primary health care level. The underlying reason for poor self-management includes both practitioner and patient-related factors. For example, practitioners in Nepal focus more on prescribing the medicines and less on supporting the development of self-management skills in the patients. In contrast, patients may not have accepted information provided by practitioners that conflicts with their own beliefs (a belief that medicines are always more effective than of lifestyle change).

In our study, the five domains of HLQ and the PAM were all found to be moderately correlated with a total score for SMPs. No previous studies have assessed the association of HL and PAM with SMPs in patients with COPD. However, studies focusing on other chronic disease have assessed these associations. In contrast to our results, a study conducted by Jacobson et al. [33] among patients with heart failure showed that self-management behaviours’ were associated with patient activation but not with health literacy. The findings of our current study are consistent with early studies that separately demonstrated a positive association between both PAM [14, 19, 33] and HL [19, 34] with self-management of behaviours. A possible reason for inconsistent results may be that different HL measures, study population and setting used in these studies.

However, neither HL nor PA is static concepts, and the finding across the studies could vary because of difference in levels of HL, health system structure, levels of understanding of their conditions and motivation to self-manage their conditions. There is a growing body of evidence showing that PA and HL are independent concepts [13, 35]. Our data confirmed that both low HL and PA were associated with inadequate engagement in SMPs.

In this community-based study, we identified that being uneducated, with a low level of family income (β = − 5.22, *p* = .002), and having one co-morbidity were all significantly associated with SMPs among the multi-morbid COPD patients of Nepal. Our finding is consistent with observations from a previous Nepalese study that concluded lack of education among the COPD patients contributed to poor SMPs such as incorrect use of inhalers [5], knowledge on self-care [6] and knowledge on COPD [7]. Patients with no education have poor functional health literacy and thus are more likely to have a poorer understanding of self-management skills including information management, symptoms management, daily lifestyle management, emotional management, and self-efficacy, in turn resulting in poor SMPs. In our study, a low level of family income was associated with poor SMPs which is supported by the findings of a review [36] that identified low-socioeconomic status as a determinant for self-management of behaviours.

Our research found a negative association between low-level SMPs and the presence of more than one co-morbidity. In contrast, a study conducted by Bringsvor et al. focusing on COPD patients that showed that the presence of co-morbidities was associated with poor self-management practices [32]. No previous research has identified an association between higher levels of SMPs with a higher number of co-morbidities. In our context, one possible explanation could be that patients being suffering from multiple morbidities may have acquired motivation and generic knowledge and self-skills that they have been able to apply across a range of conditions (e.g., physical activity, smoking cessation etc.). Moreover, patients’ having more than one co-morbidity may have sought more information on the disease and available health services using various social networks including family members, friends, relatives, school teachers, etc. A framework for health literacy and health actions developed by Wagner et al. [37] described the motivational pathway arising from the interaction between health literacy and established social cognitive variables such as health-related knowledge, attitudes, or beliefs and the decision to use primary health care and to self-manage the conditions.

Strengths of this study include a high response rate (95%), and quality of data was maintained by collecting data with the help of trained enumerators who spoke the local languages (Maithili/Tharu/Thet Maithili) of most of those in the communities studied. Limitations of the study included its cross-sectional design; which preclude definition of the cause-effect relationships. The study enrolled participants from only two rural municipalities in one district in Nepal. Thus the results may not be generalizable to a wider population and other geographical areas of Nepal. Additionally, we used self-reported questionnaires that could have some socio-desirability bias. Due to our funding constraints, we could not perform confirmatory diagnosis; instead, we selected the patients based on the patient’s medical history obtained from PHC or by looking into patients’ medical record history in the community setting.

Our study revealed a relationship between HL, PA, and other socio-demographic variables with SMPs. Good SMPs among multi-morbid COPD patients may be achieved by improving the skills of the health care professionals who deal with COPD and other chronic conditions in tailoring patient education to their health literacy levels and seeking to engage patients more actively in their care. Assessment of HL and PA in the clinical setting may help health care professionals to plan self-management support. Self-management being an effective paradigm in the prevention spectrum, delivering activities to address the medical, psychological and life style needs of patients via an integrated multi-level approach (individual, family, community and system) could be more beneficial to improve self-management behaviours. In this regard, we emphasize the need to design and implement self-management program by addressing the gaps identified by our study for improving the quality of life of patients. Furthermore, this research has opened the door for researchers who are interested in analyzing the concept of HL and PA and its incorporation in behaviour change interventions to improve SMPs and the quality of life among COPD patients and other chronic diseases.

## Conclusion

In conclusion, this study provided insights into SMPs among the multi-morbid COPD patients in a rural setting of Nepal. The findings showed that being uneducated, having a low of family income, low scores in HL and PAM are important associates that impede the SMPs in COPD patients. These findings highlight the necessity of crafting and implementing COPD self-management programs which enhance patient activation and health literacy, creating a support system for patients from low-income and low education attainment.

## Data Availability

Data is available on request from the corresponding author.

## Abbreviations

AE: Ability to find the good health information
COPD: Chronic Obstructive Pulmonary Disease
HIS: Having sufficient information to manage my own health
HL: Health Literacy
HLQ: Health Literacy Questionnaire
HPS: Feeling understood and supported by healthcare providers
HREC: Human Research Ethics Committee (HREC)
PA: Patient Activation
PAM: Patient Activation Measure
SMPQ: Self-management practices questionnaire
SMPs: Self-management practices
SS: Social support for health
UHI: Understand the health information well enough to know what to do
UNSW: University of New South Wales
